# 超高效液相色谱-四极杆-飞行时间质谱法快速筛查畜禽肉中111种农兽药

**DOI:** 10.3724/SP.J.1123.2024.10026

**Published:** 2025-09-08

**Authors:** Yiming WANG, Xiaotong LI, Kun CHU, Qianqian WANG, Shuai WU, Chen CHEN

**Affiliations:** 烟台市食品药品检验检测中心，山东 烟台 264000; Yantai Testing Center for Food and Drug，Yantai 264000，China

**Keywords:** 超高效液相色谱-四极杆-飞行时间质谱, 畜禽肉, 农兽药, 快速筛查, 多残留分析, ultra performance liquid chromatography-quadrupole-time of flight mass spectrometry （UPLC-Q-TOF/MS）, livestock and poultry meat, pesticides and veterinary drugs, rapid screening, multi-residue analysis

## Abstract

采用超高效液相色谱-四极杆-飞行时间质谱（UPLC-Q-TOF/MS）建立了畜禽肉中喹诺酮类、大环内酯类、头孢菌素类和抗病毒类等7类111种农兽药残留的数据库和定性筛查方法，实现了其中90余种化合物的定量分析。标准物质用Waters HSS T3色谱柱分离后，以甲醇和0.1%甲酸水溶液为流动相进行梯度洗脱，采用飞行时间质谱全扫描-信息关联采集-子离子扫描（TOF MS-IDA-Product Ion）模式采集，在电喷雾正离子模式下进行检测，构建111种化合物的一级精确质量数据库和二级碎片质谱库。猪肉和鸡肉样品中加入80%乙腈水溶液振荡提取，使用Oasis PRiME HLB固相萃取柱净化，在优化后的仪器条件下对方法学参数进行验证，各类化合物在相应范围内线性关系良好，线性系数（*r* ^2^）均大于0.99，方法筛查限（SDL）为0.5~10 μg/kg，有限量要求的药物均可以在其限量水平下被准确筛查。猪肉中98种化合物在1、2和10倍定量限（LOQ）加标水平下回收率为60.2%~100.2%，鸡肉中96种化合物在1、2和10倍LOQ加标水平下回收率为61.1%~116.7%，相对标准偏差（RSD）均在15%以下。基于自建质谱数据库，对市售畜禽肉样本进行筛查，结果2批次猪肉中检出恩诺沙星，1批次鸡肉中检出替米考星，检出含量为4.94~29.1 μg/kg。该方法操作简便，耗时短，为畜禽肉中农兽药多残留的高通量筛查检测提供了参考，具有实际应用价值。

## Abstract (en)

The consumption of agricultural products has increased in recent years owing to abundant production and improved living standards. Veterinary drugs are highly commercialized and widely used in animal husbandry to ensure animal health and production performance. Moreover， pesticides can become enriched during animal breeding， resulting in animal-derived food pollution through foraging， drinking， and environmental disinfection that can potentially damage human health. Consequently， food-safety issues associated with pesticide and veterinary-drug residues have attracted considerable attention. However， few reports on the multi-residue analysis of livestock and poultry meat using ultra performance liquid chromatography-quadrupole time of flight mass spectrometry （UPLC-Q-TOF/MS） have been published. Therefore， developing high-throughput and efficient screening methods for monitoring various illegal and/or restricted drugs in animal-derived foods is imperative. In this study， we developed a protocol for simultaneously examining seven pesticide and veterinary-drug types that uses an accurate mass-spectral library. UPLC-Q-TOF/MS was then employed to screen 111 such compounds， including quinolones， macrolides， cephalosporins， and antiviral drugs. The developed protocol was subsequently used to establish a method for quantitatively analyzing more than 90 compounds in livestock and poultry meat. The formula， theoretical exact mass， experimental exact mass， and retention time of each analyte were recorded and used for identification purposes. The main factors affecting the response and sensitivity of the method， such as the LC separation conditions （chromatographic column and mobile phases） and MS parameters， were optimized during instrumental analysis. Pork and chicken samples were extracted with an 80% acetonitrile aqueous solution， after which the supernatant was purified using an Oasis PRiME HLB solid-phase extraction column. The 111 target analytes were separated on a Waters HSS T3 analytical chromatographic column （100 mm×2.1 mm， 1.8 μm） after being blown with nitrogen and redissolved， with gradient elution performed using mobile phases composed of 0.1% formic acid aqueous solution and methanol. The analysis process included a flow rate of 0.4 mL/min， a column temperature of 40 ℃， and an injection volume of 5 μL， with positive electrospray ionization （ESI） and time of flight mass spectrometry full scan information-dependent acquisition-product ion （TOF MS-IDA-Product Ion） scanning modes used. The method was validated in terms of linearity， limits of screening and quantification （SDLs and LOQs， respectively）， matrix effects， accuracy， and precision. Quantification was performed using matrix-matched external-standard calibration. All target compounds exhibited good linearities in their corresponding concentration ranges， with all correlation coefficients （*r* ^2^） above 0.99. The SDLs of all analytes were in the range of 0.5-10 μg/kg， and the proportion of LOQs within the range of 0.5-10 μg/kg were 88.3% and 86.5%， respectively. The compounds quantified in pork and chicken exhibited recoveries of between 60.2% and 100.2%， and 61.1% and 116.7%， respectively， at spiked levels of LOQ， 2×LOQ and 10×LOQ， with relative standard deviations （RSDs） ranging from 1.1% to 13.9% and 1.0% to 14.1%， respectively. Simulated positive samples and commercial livestock and poultry meat samples were screened using an in-house-constructed mass spectrometry database. Commercial samples were screened while enrofloxacin was detected in two pork samples and tilmicosin was detected in one chicken sample， with content in the range of 4.94-29.1 μg/kg. The method developed in this study is advantageous because it involves simple sample processing and is less time consuming than existing methods； consequently， it is suitable for the rapid and high-throughput screening of pesticides and veterinary residues in livestock and poultry meat.

随着社会经济的发展和生活水平的提高，人们对食品的要求从需求型向质量型发生转变，食品质量安全已逐渐成为全世界关注的焦点。我国是农产品生产大国，在国际贸易中，以农兽药残留超标为代表的食品安全问题导致的贸易壁垒和纠纷影响巨大，给我国经济带来了损失^［1］^；此外，我国也是农产品消费大国，畜禽肉作为我国居民膳食结构的重要组成部分^［2］^，其品质直接关系国民的饮食健康。在养殖业的现代化和规模化过程中，兽药在治疗疾病、防止传染、改善动物源食品品质等方面起到了显著作用^［3］^，但兽药品种繁多，在利益驱使下，不规范使用乃至非法滥用现象仍然普遍存在^［4］^。此外，在畜禽动物养殖的过程中，农药可通过饲料、饮用水、圈养环境消毒等途径富集到动物体内，造成动物源性食品的污染，对人体健康带来危害^［5］^。因此，针对畜禽肉建立简便易行、快速灵敏、覆盖面广的农兽药多残留快速筛查方法具有重要的意义。

目前关于我国动物源食品中多种农兽药残留筛查的相关标准较少，已发布的标准大多针对一种或同一类别药物的检测，难以解决畜禽肉基质复杂、农兽药数量众多、化学性质差异较大等问题。传统农兽药残留的检测技术如酶联免疫分析法^［6］^、气相色谱法^［7］^和高效液相色谱法^［8，9］^等多存在检测项目单一、检测效率低、灵敏度不高、抗干扰能力差等不足。液相色谱-三重四极杆质谱法以其灵敏度高、选择性好的特点成为目前农兽药残留的主要分析方法^［10-14］^。随着高分辨质谱技术的发展，超高效液相色谱-四极杆-飞行时间质谱（UPLC-Q-TOF/MS）凭借质量精度高、采集速度快、数据可溯源和数据库可检索等优势^［15］^，越来越多地应用于农兽残筛查领域。王聪等^［16］^基于高分辨飞行时间质谱，采用QuEChERS法建立了猪肉中117种药物筛查谱库，方法检出限为1~80 μg/kg；Li等^［17］^通过优化样品前处理方法，使用C_18_固相萃取柱结合UPLC-Q-TOF/MS对猪肉中141种禁限用药物进行筛查和确证；叶磊海等^［18］^基于增强型脂质吸附剂的QuEChERS法，建立了鸡肉、牛肉、猪肝基质中32种激素药物的Q-TOF快速筛查方法。但这些方法的研究对象多为单一基质，或为不同基质中同一种类别的兽药在畜禽肉样品中同时测定多种农兽药残留鲜有报道。

Oasis PRiME HLB 净化柱是新型反相固相萃取小柱，可有效去除动物组织中蛋白质、脂肪和磷脂等基质干扰物，且使用时无需活化与平衡步骤，提取后的样品溶液可直接过柱，因此近年来被广泛用于食品基质的净化。兽药种类繁多，对人畜产生较大危害的兽药主要为抗生素类、抗病毒类、激素类和抗寄生虫类等，其中抗生素类药物按结构可分为喹诺酮类、*β*-内酰胺类、大环内酯类、酰胺醇类等，抗寄生虫类药物可分为抗线虫类、抗吸虫类和抗球虫类等。

本研究选取畜禽肉中我国消费量较大的猪肉、鸡肉两种代表性基质，以日常检验工作中发现的可能存在风险隐患的多种禁限用药物为研究对象，包括抗生素类（喹诺酮类、大环内酯类、林可酰胺类、头孢菌素类）、抗寄生虫类、抗病毒类和农药杀虫类等7类111种，采用Oasis PRiME HLB固相萃取柱净化，UPLC-Q-TOF/MS分析，建立了针对畜禽肉样品中农兽药残留的快速筛查方法。方法可在短时间内实现大批量样品中多组分药物残留的快速鉴别与分析。

## 1 实验部分

### 1.1 仪器、试剂与材料

SCIEX X500R超高效液相色谱-四极杆飞行时间质谱仪（美国 AB SCIEX公司）；BSA223S-CW型电子天平（感量0.001 g，德国赛多利斯公司）；Multi Reax型多管涡旋振荡器（德国Heidolph公司）；CR 18 RT型台式冷冻离心机（上海精科天美公司）；N-EVAP 112型氮吹仪（美国Organomation公司）；Milli-Q型超纯水机（美国Millipore公司）。

111种农兽药标准物质（包括29种喹诺酮类、16种大环内酯类、3种林可酰胺类、18种头孢菌素类、22种抗寄生虫类、7种抗病毒类和16种农药杀虫类，具体见表1）分别购于天津阿尔塔科技有限公司、农业农村部环境保护科研监测所和上海安谱实验科技股份有限公司，其中18种头孢菌素类化合物、吡哌酸和依托地红霉素为纯度≥85%的固体标准物质，其余91种标准品为质量浓度100~1 000 μg/mL的液体标准溶液；Oasis PRiME HLB固相萃取柱（6 mL/200 mg，美国沃特世公司）；甲酸、甲醇、乙腈（色谱纯，德国Merck公司）；0.22 μm聚四氟乙烯（PTFE）滤膜（美国安捷伦公司）；猪肉和鸡肉样品（超市购买）。

**表 1:** 111种化合物的保留时间、质谱参数及在猪肉和鸡肉中的筛查限（SDL）和定量限（LOQ）

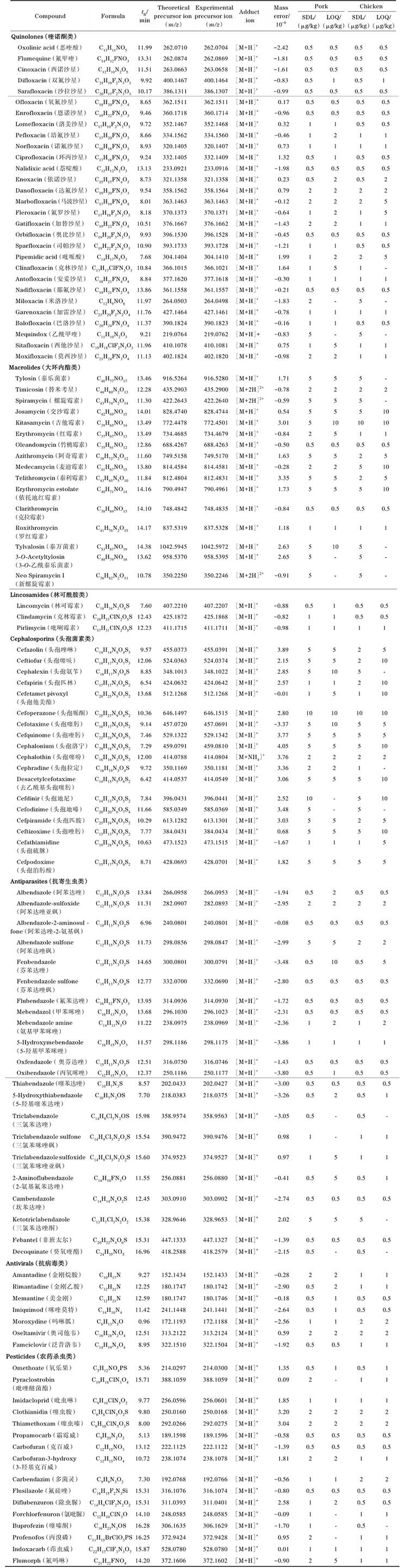

### 1.2 标准溶液的配制

固体标准物质根据溶解度选择不同的溶解试剂。分别准确称取吡哌酸和依托地红霉素标准物质，用甲醇配制成1 000 μg/mL的标准储备溶液；准确称取18种头孢菌素类标准物质，以乙腈-水混合溶液（1∶3，v/v）为溶剂配制成100 μg/mL的混合标准储备溶液，在-18 ℃下避光保存。配制混合标准溶液时，首先按照不同类别配制成10 μg/mL的混合标准工作溶液，使用时以50%甲醇水溶液配制成111种100 ng/mL的混合标准工作溶液。

### 1.3 样品前处理

取适量样品绞碎均质，-18 ℃以下保存。实验前提前取出，恢复至室温后使用。称取2.00 g均质后的样品，加入8 mL 80%乙腈水溶液，振荡20 min，10 000 r/min离心5 min，取5 mL上清液过Oasis PRiME HLB固相萃取净化柱，净化柱无需活化、洗脱等步骤，收集全部流出液，准确移取4 mL于试管中，40 ℃氮气吹干，加入1 mL 50%甲醇水溶液涡旋混匀，过0.22 μm PTFE滤膜，待上机分析。

### 1.4 色谱条件

Waters HSS T3色谱柱（100 mm×2.1 mm， 1.8 μm）；柱温：40 ℃；流速：0.3 mL/min；进样量：5 μL；流动相：A相为0.1%甲酸水溶液，B相为甲醇；梯度洗脱程序：0~1 min，5%B；1~8 min，5%B~30%B；8~11 min，30%B~50%B；11~16 min，50%B~95%B；16~17 min，95%B；17~17.1 min，95%B~5%B；17.1~19 min，5%B。

### 1.5 质谱条件

离子源：电喷雾离子源（ESI）；AB SCIEX自动校正系统（CDS）：每6个样品自动校正一次，校正液流速0.05 mL/min；扫描模式：正离子扫描；离子化电压：5 500 V；离子源温度：550 ℃；雾化气流量：55 L/h；气帘气流量：35 L/h；辅助气流量：55 L/h。设置母离子强度大于100 cps时启动信息依赖型子离子采集，TOF-MS参数：扫描范围*m/z* 100~1 100，去簇电压80 V，碰撞能量10 eV。TOF-MS/MS参数：扫描范围*m/z* 50~1 000，去簇电压80 V，碰撞能量（35±15） eV。

### 1.6 数据库的建立

#### 1.6.1 一级精确质量数据库

在Library View数据库软件中输入111种农兽药化合物的名称、分类及分子式，将质量浓度为100 ng/mL的标准溶液进行一级质谱全扫描，获得每种目标物的保留时间、母离子精确质量数、质量偏差和离子化形式等信息，完成一级精确质量数据库的创建。

#### 1.6.2 二级碎片离子数据库

设置飞行时间质谱全扫描-信息关联采集-子离子扫描（TOF MS-IDA-Product Ion）采集模式，当目标化合物的响应值超过阈值时，得到不同碰撞能量产生的主要碎片离子的精确质量数和谱图，将采集到的二级谱图添加至Library View中建立目标化合物的二级谱库，与相应的名称、保留时间、精确质量数测定值等信息关联，完成谱库构建，化合物信息见表1。

## 2 结果与讨论

### 2.1 色谱条件的优化

#### 2.1.1 色谱柱的选择

目标分析物种类广泛，理化性质各异，涉及多组精确质量数相似或同分异构体化合物，为了最大限度地兼顾所有组分的分离度和响应值，需要对色谱条件和质谱条件逐一进行优化，从而实现111种化合物的同时分析。本实验比较了不同品牌、不同粒径的4种反相色谱柱Waters HSS T3（100 mm×2.1 mm，1.8 μm）、Waters BEH C18（100 mm×2.1 mm，1.7 μm）、Agilent Eclipse Plus C18（100 mm×2.1 mm，3.5 μm）和Phenomenex Kinetex F5（100 mm×2.1 mm，2.6 μm）对目标化合物（50 ng/mL）出峰情况的影响。结果表明，大部分化合物在Waters HSS T3色谱柱上的灵敏度更高，分离效果更好，尤其是头孢菌素类药物在Waters HSS T3柱上的响应值显著高于其他3种色谱柱，结果见图1。

**图1:**
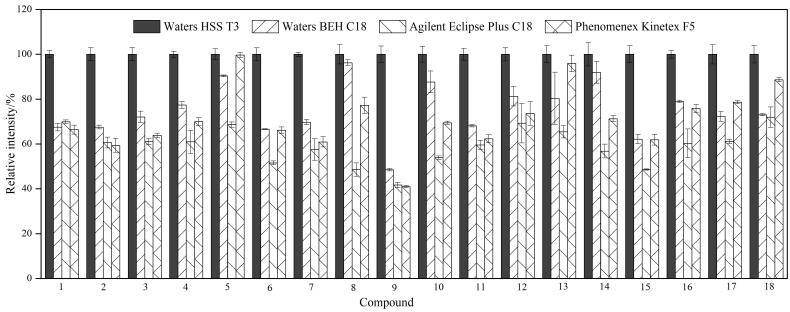
不同色谱柱对18种头孢菌素类化合物响应值的影响（*n*=3） Compounds： 1. cefazolin； 2. ceftiofur； 3. cephalexin； 4. cefapirin； 5. cefetamet pivoxyl； 6. cefoperazone； 7. cefotaxime； 8. cefquinome； 9. cephalonium； 10. cephalothin； 11. cephradine； 12. desacetylcefotaxime； 13. cefdinir； 14. cefodizime； 15. cefpiramide； 16. ceftizoxime； 17. cefathiamidine； 18. cefpodoxime.

#### 2.1.2 流动相的选择

为了进一步使色谱峰分离且获得良好峰形、提高目标化合物的灵敏度，实验对流动相体系进行了优化，考察了水、0.1%甲酸水溶液、2 mmol/L乙酸铵溶液、2 mmol/L乙酸铵溶液（含0.1%甲酸）与甲醇或乙腈不同组合作为流动相时各目标物（50 ng/mL）的分离效果。

甲醇-水和乙腈-水作为流动相时，目标物色谱分离度差，突出表现在喹诺酮类药物色谱峰峰宽且对称性差。为改善色谱峰形，在流动相的水相中添加2 mmol/L乙酸铵缓冲盐，色谱峰形一定程度上得到改善，但依然表现出峰宽较大、分离度差的问题。在水相中添加甲酸后，目标化合物的峰形集中变窄，离子化效率提高，化合物响应提高^［19］^。对比0.1%甲酸水溶液-甲醇体系和0.1%甲酸水溶液-乙腈体系，结果发现，有机相为甲醇时大多数目标化合物的灵敏度高于乙腈体系（仅泰乐菌素、螺旋霉素、交沙霉素、吉他霉素、泰万菌素、3-*O*-乙酰泰乐菌素和新螺旋霉素等7种大环内酯类化合物在乙腈体系中响应优于甲醇体系），此外，乙腈的洗脱能力较强^［20］^，多种化合物在乙腈体系下会出现肩峰或无法达到完全基线分离的情况，图2为3-羟基克百威和氨基甲苯咪唑在0.1%甲酸水溶液-乙腈和0.1%甲酸水溶液-甲醇体系中的提取离子色谱图。综合考虑，选择0.1%甲酸水溶液-甲醇为流动相。图3为50 ng/mL的111种化合物混合标准溶液的提取离子色谱图。

**图2:**
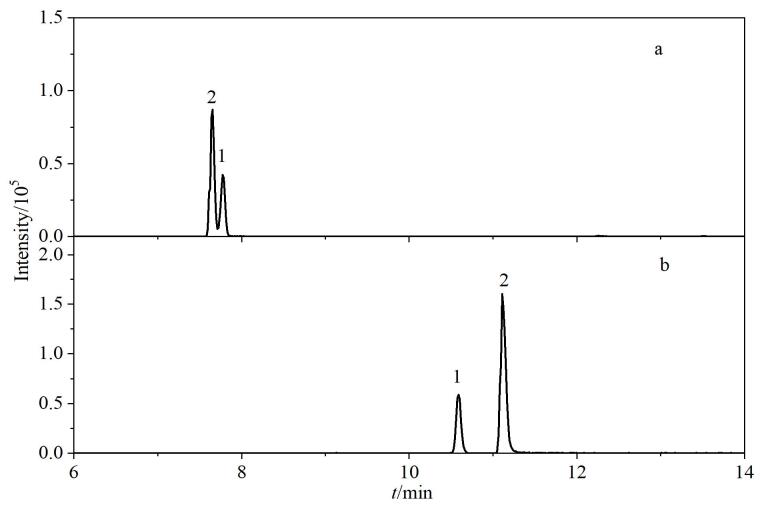
3-羟基克百威和氨基甲苯咪唑在不同流动相下的提取离子色谱图 a. acetonitrile-0.1% formic acid aqueous solution； b. methanol-0.1% formic acid aqueous solution. Peak identifications： 1. carbofuran-3-hydroxy； 2. mebendazole amine.

**图3:**
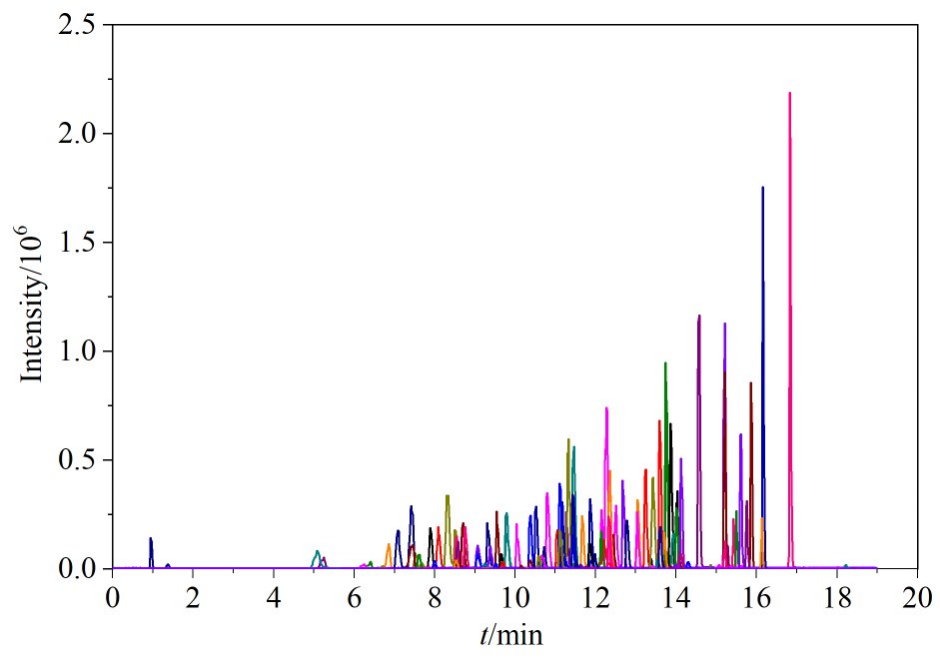
111种化合物的提取离子流色谱图

### 2.2 质谱条件的优化

#### 2.2.1 扫描方式的选择

根据待测化合物的结构特征，选择正、负离子两种模式进行扫描，结果表明111种化合物在正离子模式下的响应较高。通过对比化合物不同加合离子的灵敏度，如［M+H］^+^、［M+NH_4_］^+^、［M+Na］^+^等，选择响应值最高的离子作为加合物离子。实验发现大多数化合物的前体离子为质子化［M+H］^+^分子离子，但头孢噻吩的［M+NH_4_］^+^准分子离子峰响应比［M+H］^+^峰高；大环内酯类化合物中替米考星、螺旋霉素和新螺旋霉素除了形成［M+H］^+^，还形成［M+2H］^2+^，且［M+2H］^2+^的峰响应比［M+H］^+^的响应高，各化合物的具体离子加合方式见表1。

#### 2.2.2 质谱参数的优化

为了获得丰富可靠的实验数据，本研究利用仪器的信息依赖型子离子采集模式，建立TOF MS-IDA-Product Ion工作流程，对目标化合物进行TOF-MS-IDA扫描，获得目标物的一级质谱信息，在进行一级质谱扫描的同时，对满足触发二级的离子进行TOF-MS/MS扫描，获得离子的二级碎片信息。通过增加扩展碰撞能量获取高质量区和低质量区的质谱碎片，使每种化合物所建立的二级质谱库更加精准。经过实验优化，结合各种待测化合物的仪器响应情况，设置碰撞能量为35 eV，扩展碰撞能量为15 eV，当目标化合物的响应值超过IDA阈值（100 cps）时，可获得由不同碰撞能量（20、35、50 eV）叠加组成的二级碎片谱图，同时开启仪器的动态背景扣除功能，可极大降低本底背景的二级质谱信号强度，提高样品中目标物的响应。

#### 2.2.3 基于谱库的筛查与鉴定

将添加了111种目标化合物的畜禽肉样品进行质谱扫描测定，对分子离子的保留时间、精确质量偏差、同位素丰度比和二级碎片离子等信息进行综合分析，对于二级碎片离子与数据库匹配得分值大于70的化合物进行确证分析^［21］^。以西诺沙星为例，IDA模式下采集的提取离子色谱图、一级同位素质谱图、二级质谱图及数据库匹配结果见图4。

**图4:**
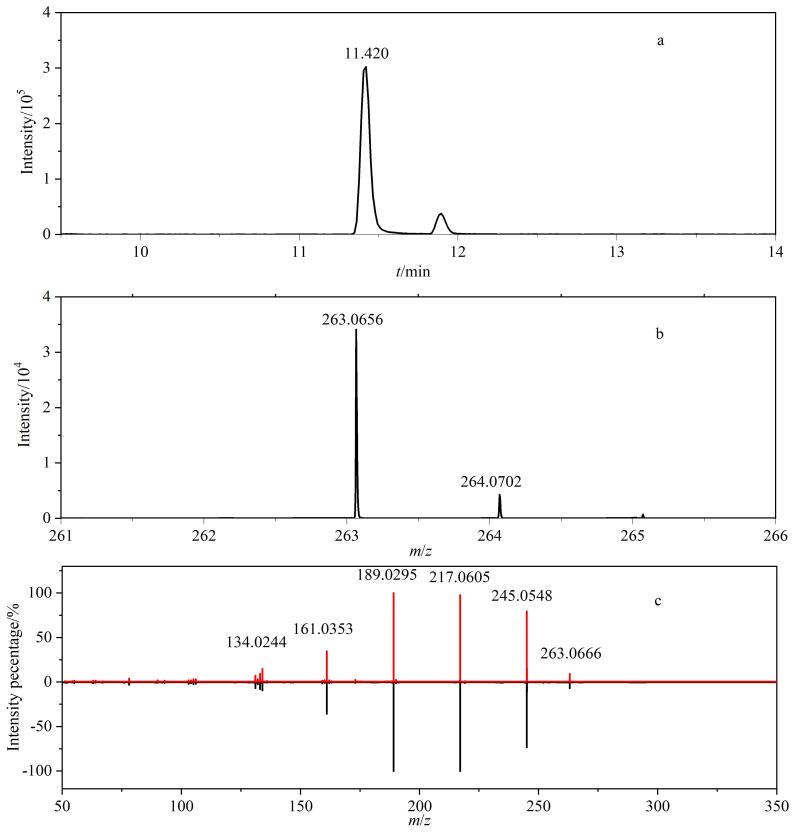
西诺沙星的（a）提取离子色谱图、（b）一级同位素质谱图和（c）二级质谱图

### 2.3 方法学评价

#### 2.3.1 基质效应

基质效应（ME）是质谱检测中普遍存在的现象，通常采用基质标准曲线斜率与溶剂标准曲线斜率的比值来评价基质效应。ME值为80%~120%时，表明基质效应不明显；ME值超过120%时，表明存在基质增强效应；ME值低于80%时，则存在基质抑制效应。不同样品产生的基质效应可能会不同，本实验分别考察了猪肉和鸡肉的基质效应，结果表明两种基质中分别有76种和61种化合物表现为基质抑制，因此，为降低基质抑制对化合物的影响，采用空白基质标准曲线进行检测。

#### 2.3.2 筛查限

参照欧盟EU/2021/808^［22］^规定和仝凯旋等^［23］^的定性验证方法，将目标物在至少95%的样本中被检测到的最低水平定义为该化合物在该方法中的筛查限。在25份猪肉和鸡肉空白样品中分别添加混合标准溶液至5个水平（0.5、1、2、5、10 μg/kg），经过前处理后进行筛查检测，通过对筛查结果的分析确定111种农兽药化合物的SDL。实验结果如表1所示，猪肉和鸡肉样品中化合物的SDL集中在0.5~5 μg/kg，其中SDL≤2 μg/kg的化合物分别占76.6%和80.2%。

111种化合物中，我国兽药限量标准GB 31650-2019^［24］^和GB 31650.1-2022^［25］^对猪肉和鸡肉基质有限量要求的化合物分别为24种和21种，限量范围为2~1 000 μg/kg，本方法可以很好地满足畜禽肉中有限量要求的药物的筛查需求。

#### 2.3.3 线性范围、线性关系与定量限

用空白基质提取液配制质量浓度为0.2~100 ng/mL的基质混合标准工作溶液，在优化条件下进行测定，根据色谱峰面积与分析物的质量浓度建立标准曲线，各化合物在相应质量浓度范围内线性关系良好，相关系数（*r* ^2^）均大于0.99。在阴性样品中添加目标化合物，经过前处理后以满足GB 5009.295-2023^［26］^中回收率和精密度要求的最低添加浓度作为方法的定量限（LOQ），结果如表1所示。猪肉和鸡肉中分别有13种和15种化合物的回收率小于60%，如米洛沙星、乙酰甲喹、三氯苯达唑、新螺旋霉素、头孢地嗪等，只能进行定性分析。猪肉和鸡肉基质中分别有98种和96种化合物的LOQ为0.5~10 µg/kg，其中LOQ≤5 μg/kg的化合物分别占93.9%和87.5%。将本方法验证的部分目标化合物与文献进行对比，对于喹诺酮类和大环内酯类化合物，文献［^16^］中方法的检出限（LOD）范围为1~60 µg/kg，本方法的LOQ为0.5~5 µg/kg，表明本方法可用于畜禽肉中药物残留的测定。

#### 2.3.4 回收率与精密度

对猪肉和鸡肉样品中可定量分析的90余种化合物在1、2和10倍LOQ 3个添加水平下进行加标回收试验，每个添加水平平行测定6次，计算每种化合物的加标回收率和相对标准偏差（RSD）。结果表明，猪肉样品中化合物在3个加标水平下的平均回收率分别为60.2%~94.9%、62.7%~98.5%和61.3%~100.2%，RSD分别为1.4%~12.2%、1.1%~13.4%和1.8%~13.9%；鸡肉样品中化合物在3个加标水平下的平均回收率分别为62.1%~116.7%、61.1%~112.9%和63.5%~108.6%，RSD分别为1.2%~11.0%、1.6%~14.1%和1.0%~13.5%。3个加标水平下分布在60%~70%和70%~120%范围的化合物个数相同，其中猪肉和鸡肉基质中回收率在70%~120%范围内的待测物占比分别为76.5%和83.3%，统计结果如表2所示。

**表2:** 猪肉和鸡肉中化合物在不同回收率范围的个数统计

Compounds	Pork		Chicken	
	60%-70%	70%-120%	60%-70%	70%-120%
Quinolones	6	21	4	22
Macrolides	5	9	3	8
Lincosamides	0	3	0	3
Cephalosporins	2	14	3	12
Antiparasites	7	12	4	15
Antivirals	1	6	0	7
Pesticides	2	10	2	13

### 2.4 方法应用

#### 2.4.1 模拟加标样本分析

通过本实验建立的方法对加标水平为10 μg/kg的样品进行筛查验证。样品经前处理后测试，结果显示待测样品中111种目标化合物的精确质量数偏差绝对值均小于5×10^-6^，保留时间偏差为±2.5%，与高分辨质谱数据库的质谱信息匹配一致，符合确证要求。

#### 2.4.2 实际样本检测

采用所建立的方法对市售的60批次畜禽肉样品进行检测。实验结果表明，2批次猪肉中检出恩诺沙星，含量分别为16.2 μg/kg和29.1 μg/kg；1批次鸡肉中检出替米考星，含量为4.94 μg/kg。检出的化合物含量处于较低水平，均未超出GB 31650-2019规定的限量。

## 3 结论

本研究采用UPLC-Q-TOF/MS结合PRiME HLB固相萃取柱建立了111种农兽药残留药物筛查数据库，针对猪肉和鸡肉基质进行筛查方法的开发和验证，并将该方法应用于市售畜禽肉中农兽药残留筛查。该方法前处理过程简单、快速，通量大，准确性高，适用于畜禽肉中多种常见农兽药残留组分的同时快速检测，可满足突发食品安全事件快速定性的检测要求，为动物源基质中的农兽药筛查检测提供了可靠的技术支撑。
